# Antegrade bilateral metal stent deployment under endoscopic ultrasound guidance for hepatic hilar obstruction using locking stent technique

**DOI:** 10.1055/a-2556-6375

**Published:** 2025-03-21

**Authors:** Takeshi Ogura, Yuki Uba, Nobuhiro Hattori, Kimi Bessho, Hiroki Nishikawa

**Affiliations:** 1 38588 Endoscopy Center, Osaka Medical and Pharmaceutical University Hospital, Osaka, Japan; 2 13010 2nd Department of Internal Medicine, Osaka Medical and Pharmaceutical University, Osaka, Japan

Endoscopic ultrasound-guided biliary drainage (EUS-BD) has been widely performed for distal biliary obstruction. Due to improvements in devices and techniques, EUS-BD has recently been indicated for hepatic hilar biliary obstruction (HBO). In cases of surgically altered anatomy, right hepatic biliary drainage using the bridging technique is needed to perform bilateral biliary drainage because the access route is from the left hepatic bile duct 1 . After the bridging technique, a metal stent should be deployed at the periphery of the bile duct to obtain sufficient drainage area. However, if metal stent deployment at the periphery of the bile duct is performed, stent dislocation can occur because the length of the metal stent is short in the hepatic site 2 3 . To prevent this adverse event, a sufficient length of stent in the hepatic site should be obtained. Technical tips for EUS-BD for HBO using the previously described locking stent technique 4 to prevent stent dislocation and obtain sufficient drainage area are described.

An 80-year-old woman was admitted to our hospital due to obstructive jaundice. On computed tomography (CT), a hepatic hilar tumor with liver metastasis was observed. She was diagnosed with unresectable hepatic hilar cholangiocarcinoma by liver biopsy. Because of duodenal stenosis due to cholangiocarcinoma, a duodenoscope could not be advanced into the duodenum. Therefore, EUS-BD was attempted. First, the intrahepatic bile duct was punctured using a 19-G needle, and contrast medium was injected. After obtaining a cholangiogram, an HBO was observed. A 0.025-inch guidewire was successfully advanced into the right hepatic site ( Fig. 1 ). To adjust the straight angle of the guidewire between the left and right hepatic bile ducts, the double-guidewire technique was performed using an uneven endoscopic retrograde cholangiopancreatography (ERCP) catheter ( Fig. 2 ). Then, an uncovered metal stent was deployed from the right to the left hepatic bile duct ( Fig. 3 ). Next, the guidewire was inserted into the common bile duct through the mesh of the uncovered metal stent, and an uncovered metal stent was deployed from the common bile duct to the left hepatic bile duct using the stent-in-stent technique ( Fig. 4 ). To prevent bile duct branch obstruction and obtain sufficient drainage area, the distal end of the partially covered metal stent was placed within the first and second uncovered metal stents (locking stent technique) and successfully deployed ( Fig. 5 , Video 1 ). No adverse events, including focal cholangitis or stent dislocation, were observed during the 280-day follow-up.

**Fig. 1:**
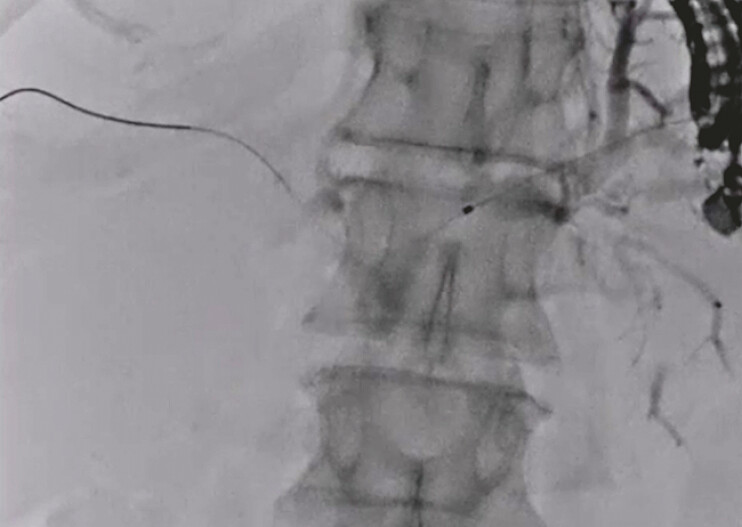
A 0.025-inch guidewire is successfully advanced into the right hepatic site.

**Fig. 2:**
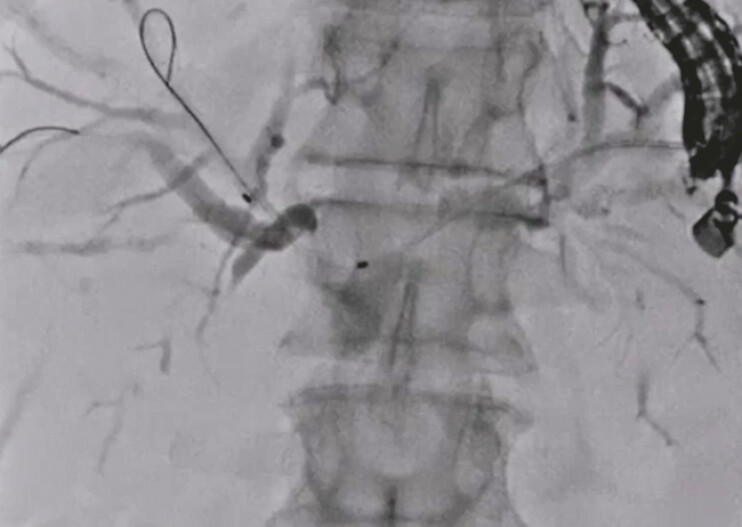
The double-guidewire technique is performed using an uneven endoscopic retrograde cholangiopancreatography catheter.

**Fig. 3:**
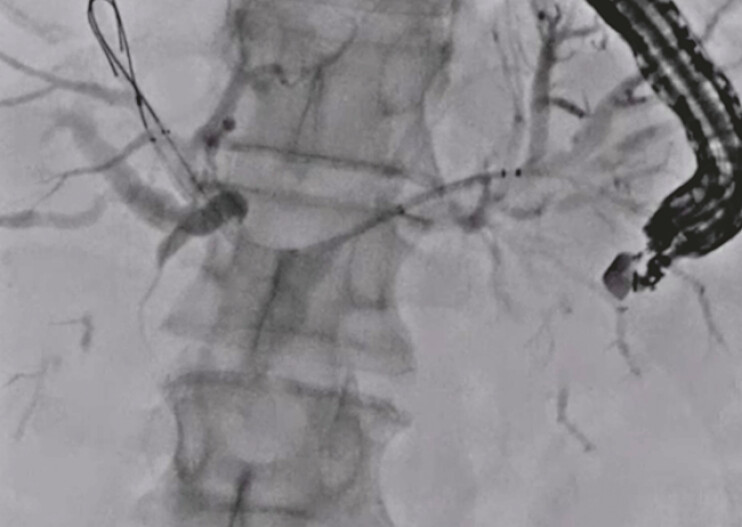
An uncovered metal stent is deployed from the right to the left hepatic bile duct.

**Fig. 4:**
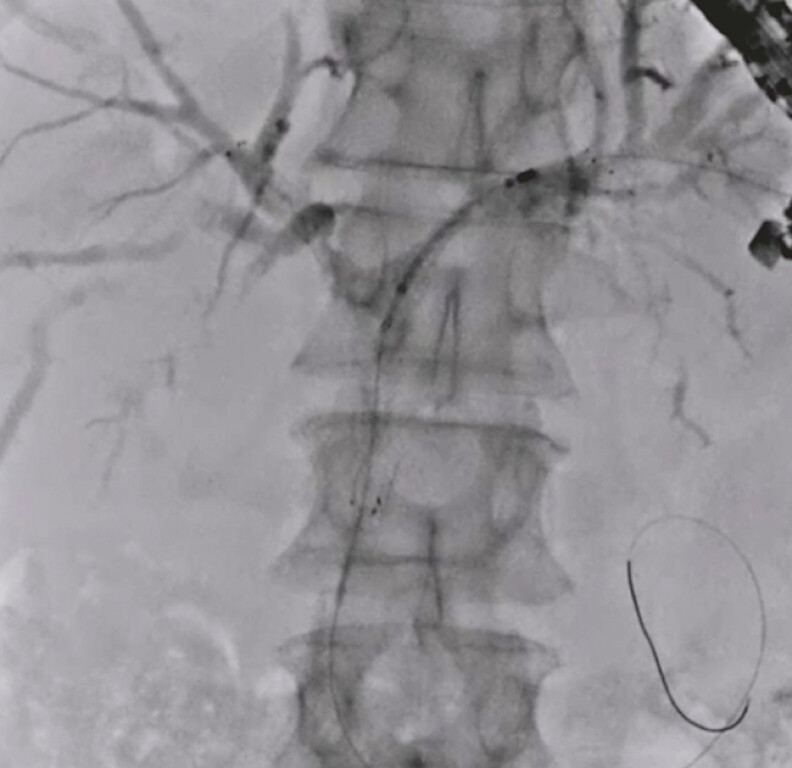
An uncovered metal stent is deployed from the common bile duct to the left hepatic bile duct using the stent-in-stent technique.

**Fig. 5:**
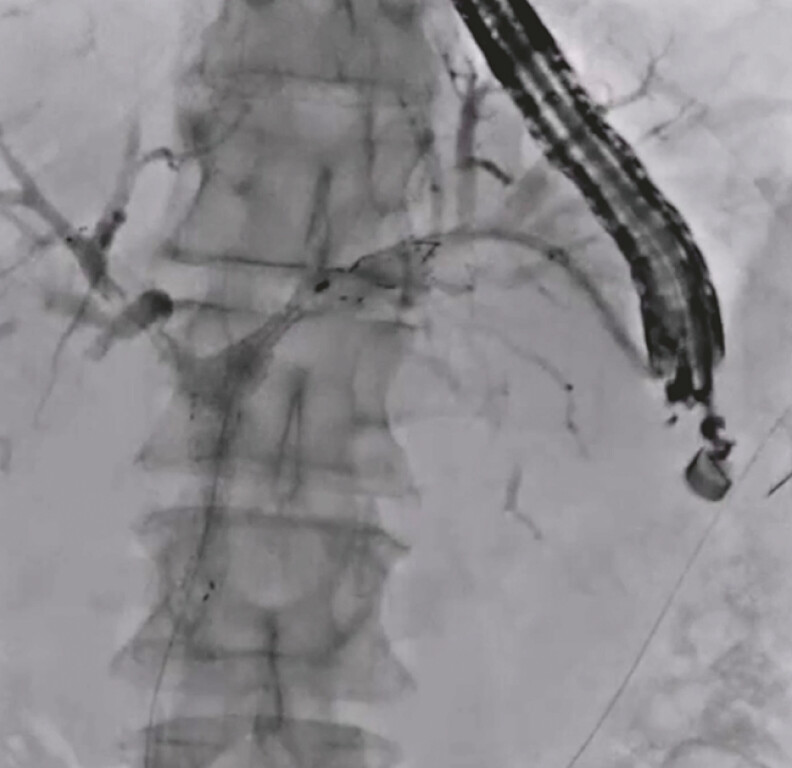
The distal end of the partially covered metal stent is placed within the first and second uncovered metal stents and successfully deployed.

The locking stent technique is performed under endoscopic-ultrasound guidance for hepatic hilar obstruction.Video 1

In conclusion, the locking stent technique may be useful in EUS-BD for HBO to obtain sufficient biliary drainage area and prevent stent dislocation.

Endoscopy_UCTN_Code_TTT_1AS_2AH

## Notes

Endoscopy E-Videos https://eref.thieme.de/e-videos
*E-Videos* is an open access online section of the journal *Endoscopy* , reporting on interesting cases and new techniques in gastroenterological endoscopy. All papers include a high-quality video and are published with a Creative Commons CC-BY license. Endoscopy E-Videos qualify for HINARI discounts and waivers and eligibility is automatically checked during the submission process. We grant 100% waivers to articles whose corresponding authors are based in Group A countries and 50% waivers to those who are based in Group B countries as classified by Research4Life (see: https://www.research4life.org/access/eligibility/ ). This section has its own submission website at https://mc.manuscriptcentral.com/e-videos .
